# Prospective Analysis of the Impact of ^68^Ga-DOTATOC Positron Emission Tomography–Computerized Axial Tomography on Management of Pancreatic and Small Bowel Neuroendocrine Tumors

**DOI:** 10.1097/MPA.0000000000001625

**Published:** 2020-08-07

**Authors:** Silvia N. Ghobrial, Yusuf Menda, Gideon K. Zamba, Sarah L. Mott, Kristin Gaimari-Varner, David Dick, Joseph Dillon, James R. Howe, Michael Graham, John Sunderland, Andrew Bellizzi, Thomas M. O’Dorisio, M. Sue O’Dorisio

**Affiliations:** From the Departments of ∗ Pediatrics; † Radiology; ‡ Biostatistics; § Holden Comprehensive Cancer Center; Departments of ∥ Medicine; ¶ Surgery; # Pathology, University of Iowa, Iowa City, IA.

**Keywords:** ^68^Ga-DOTATOC, PET-CT, neuroendocrine tumor, change in management

## Abstract

**Objectives:**

A prospective clinical trial evaluated the effect of ^68^Ga-DOTATOC positron emission tomography–computerized axial tomography (PET-CT) on change in management of patients with lung, pancreatic, and small bowel neuroendocrine tumors. The primary eligibility criterion was a histologically proven tumor with positive somatostatin receptor subtype 2A immunohistochemistry. The primary and secondary end points were change in patient management and safety.

**Methods:**

Referring physicians completed questionnaires pre– and post–^68^Ga-DOTATOC PET-CT, stating current and planned patient management, respectively, with tumor board adjudication of final management decisions. Change in management was categorized as follows: no change; minor change (additional imaging, supportive care); or major change (octreotide/lanreotide therapy, tumor biopsy, surgery, peptide receptor radiotherapy, chemotherapy, biological therapy, liver embolization).

**Results:**

A major change in management was recommended for 54 (47.37%) of 114 subjects and a minor change for 6 (5.26%) of 114 subjects, with no change for 54 (47.37%) of 114 subjects. Grade 1 adverse events were observed in 26 of 114 subjects (nausea, headache, back pain, diarrhea); one grade 2 (petechiae) and one grade 3 (abdominal pain) adverse event were observed. No grade 2 or 3 adverse events were related to study drug and none required intervention.

**Conclusions:**

Imaging with ^68^Ga-DOTATOC PET-CT has a significant impact on management of patients with neuroendocrine tumors.

Neuroendocrine tumors (NETs) are solid tumors with malignant potential that arise from precursor cells in the endocrine system, including intestines, pancreas, lungs, ovaries, thyroid, pituitary, and adrenal glands. Most NETs are slow-growing, well-differentiated grade 1, 2, or 3 tumors, whereas some are aggressive, poorly differentiated grade 3 tumors.

The clinical behavior of NETs is extremely variable; they exhibit a wide range of clinical behaviors because of the episodic secretion of neuropeptide hormones such as insulin, serotonin, glucagon, gastrin, and vasoactive intestinal peptide. Symptoms are usually nonspecific but can be debilitating, including diarrhea, bronchospasm, flushing, and cardiac valve disease.^1^

The incidence of NETs has been steadily rising for the past decade, in part because of earlier detection. The age-adjusted incidence rate increased from 1.09/100,000 in 1973 to 6.98/100,000 in 2012, and the prevalence rate has markedly increased to 171,321 in 2014.^2^ Diagnosis and staging of these tumors are challenging and require a multimodality approach. Neuroendocrine tumors characteristically express somatostatin receptor 2A (SST2A) that can be exploited as a diagnostic target. Before 2015, Octreoscan single-photon emission tomography (SPECT) along with multiphase contrast-enhanced CT or magnetic resonance imaging was standard of care for diagnosis and staging of NETs.^3^ Positron emission tomography (PET) is more sensitive than SPECT; the Food and Drug Administration (FDA) approved ^68^Ga-DOTATATE as a PET tracer in 2016. The goal in this study was to prospectively evaluate whether or not ^68^Ga-DOTATOC, newly FDA approved as a PET imaging agent in 2019, would have a significant impact on patient management.

## MATERIAL AND METHODS

This study was a prospective, phase 2, open-label trial conducted at the University of Iowa. The primary end point was the change in management of patients with known or suspected NETs and other somatostatin receptor-positive tumors based on findings of the ^68^Ga-DOTATOC PET-CT scan. Participants were recruited from the NET clinic at the University of Iowa Holden Comprehensive Cancer Center, the University of Iowa Stead Family Children's Hospital, and via clinicaltrials.gov (Identifier: NCT02441062). The study was approved by the University of Iowa Institutional Review Board, and all subjects signed written consent to participate in the trial.

Eligible participants were enrolled for suspected disease progression, restaging before initiation of new therapy, measurement of disease response after therapy, evaluation of unknown primary, or suspected but undiagnosed NET. ^68^Ga-DOTA0-Tyr3-octreotide (^68^Ga-DOTATOC) was prepared and administered under an FDA-approved investigator's new drug (IND #114398) agreement (now FDA-approved as NDA #210828). Study participants received 3 to 5 mCi ^68^Ga-DOTATOC and 50 to 70 minutes later underwent a PET-CT scan on a Siemens Biograph 40 PET-CT (Seimens AG, Munich, Germany) with PET imaging in 3-D mode; a low-dose CT without contrast was acquired for attenuation correction and anatomical correlation. Reconstructed PET, CT, and fused PET-CT images were reviewed on computer monitor in coronal, sagittal, and transverse planes. Patients remained in the imaging facility for at least 2 hours from the time of radiopharmaceutical injection and were contacted by phone 24 hours later to monitor for adverse events, which were graded according to NCI Common Terminology Criteria version 4.0. (CTCAE v4.0).

Referring physicians were asked to fill out a pre-PET scan form to provide information on the management and treatment plan for the patient before the ^68^Ga-DOTATOC PET-CT. After official reading of the ^68^Ga-DOTATOC PET-CT was available, the treating physician was asked to fill out a post-PET scan form indicating the management and treatment plan recommended to the patient. Change in management strategy criteria of the National Oncologic PET Registry study were modified for the specific treatment strategies used in NET.^4^ Three level of changes were assessed: no change, minor change, and major change (Table 1). All major changes were adjudicated by a multidisciplinary tumor board including board-certified physicians in surgery, nuclear medicine, internal medicine, pediatrics, and pathology.

**TABLE 1:**
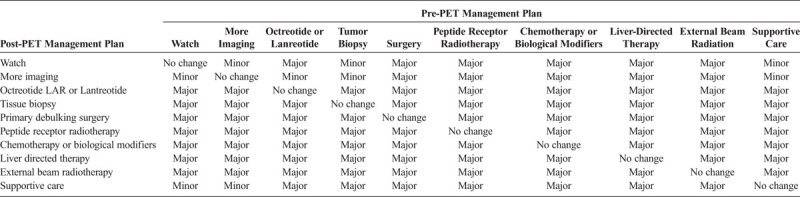
Criteria for Classification of Changes in Treatment Plan Based on ^68^Ga-DOTATOC PET-CT

## RESULTS

**FIGURE 1:**
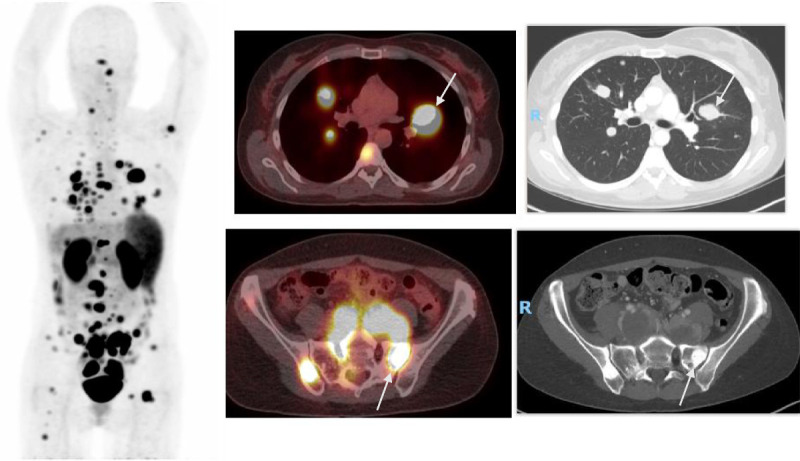
Major change: treatment with PRRT. Multiple ^68^Ga-DOTATOC avid lung lesions, extensive lymphadenopathy, and multiple sclerotic bone lesions on whole body and fused PET-CT and CT images. Representative transaxial CT slices show bilateral lung nodules and bilateral sclerotic iliac bone lesions (arrows on representative lesions) with intense uptake (SUVmax up to 29.5) on fused PET-CT image at the same level. Major change recommendation: PRRT.

**FIGURE 2:**
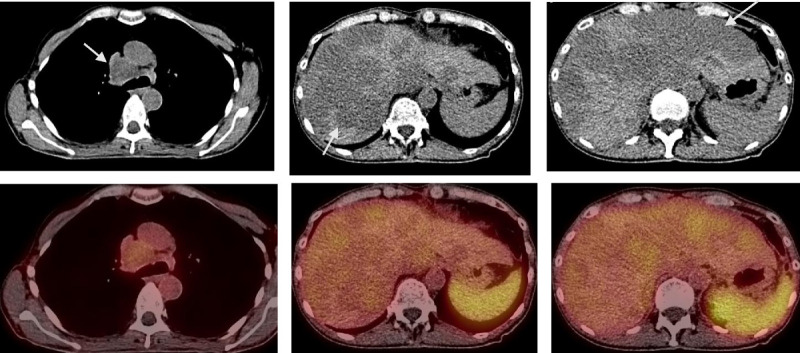
Major change: chemotherapy or biologic therapy. Computed tomography and fused ^68^Ga-DOTATOC PET-CT images through mediastinal nodes (arrow in left column) and through the liver (arrows in mid and right columns). Neither large mediastinal node nor hypodense liver lesions show uptake of ^68^Ga-DOTATOC. Patient is not considered a candidate for PRRT.

One hundred fourteen subjects, aged 14 months to 91 years, 45% female, underwent a ^68^Ga-DOTATOC PET-CT. A major change in management was recommended for 54 (43.37%) of the 114 subjects, minor change for 6 subjects (5.26%), and no change for 54 subjects (43.37%), with 95% exact confidence intervals of 37.9% to 56.9%, 2.0% to 11.1%, and 37.9% to 56.9%, respectively (Table 2). The most frequent recommendation for major change (14/54 subjects) was to treat with peptide receptor radiotherapy (PRRT) as indicated in Table 3. An example of the previously unrecognized need for PRRT is illustrated in Figure 1. The ^68^Ga-DOTATOC PET-CT for this subject demonstrated multiple osseous and lymph node metastatic lesions that were not appreciated on conventional imaging. Figure 2 demonstrates a subject in whom lesions seen on conventional imaging were shown by ^68^Ga-DOTATOC PET-CT to be SST2A negative; consequently, chemotherapy or biologic agents were recommended rather than PRRT.

**TABLE 2:**
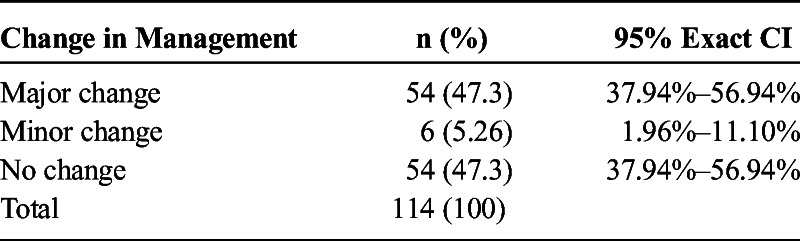
Frequency of Changes in Care Management Following ^68^Ga-DOTATOC PET-CT

**TABLE 3:**
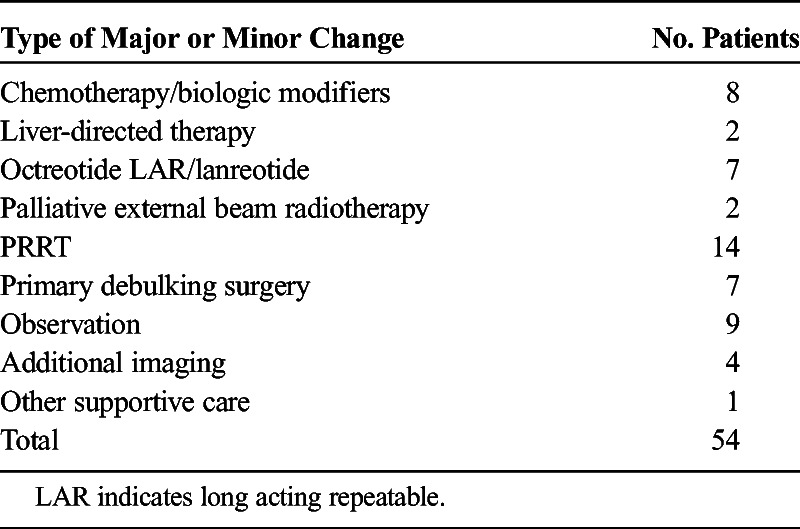
Recommendations of Multidisciplinary Tumor Board Based on ^68^Ga-DOTATOC PET-CT

Among the 114 subjects enrolled, 26 experienced a grade 1 adverse event (AE), 11 of which were possibly related to ^68^Ga-DOTATOC. One grade 2 AE (petechiae) and one grade 3 AE (abdominal pain) were observed, neither of which was related to study drug.

## DISCUSSION

Positron emission tomography radiopharmaceuticals labeled with gallium-68 (^68^Ga-) have been developed for several somatostatin analogs including ^68^Ga-DOTA^0^-Tyr^3^-octreotide (^68^Ga-DOTATOC); ^68^Ga-DOTA^0^-1NaI^3^-octreotide (^68^Ga-DOTANOC); and ^68^Ga-DOTA^0^-Tyr^3^-octreotate (^68^Ga-DOTATATE). ^68^Ga-DOTATOC and ^68^Ga-DOTATATE PET-CT have significant advantages over Octreoscan SPECT-CT imaging in view of higher affinity for SST2A, lower radiation dose (3.4 mSv vs 12 mSv) and more convenient for patients, requiring a single visit of 3 to 4 hours rather than 2 visits for 24 hours.

Early retrospective studies demonstrated the impact of ^68^Ga-DOTATOC in the management of patients with NETs. A systematic review and meta-analysis of these studies summarized the impact of ^68^Ga-DOTATOC in patient management. A combined 188 subjects with histologically proven NETs were imaged using ^68^Ga-DOTATOC PET-CT across 4 independent studies. An overall 51% change in management was reported.^5^ Unfortunately, retrospective studies can be compromised because of selection bias, loss of follow-up, and potential underestimation of changes in management that were not clearly documented in patients' charts.

This prospective study included 114 subjects; major and minor changes in management were clearly defined before beginning the study. A care plan based on conventional imaging and available pathologic findings was documented by the referring physician in a prescan questionnaire filed in the subject's source data before the ^68^Ga-DOTATOC PET-CT. Every PET-CT was interpreted independently by 2 nuclear medicine physicians and the consensus report filed in the patient's medical record as well as in the source data for the trial. All major changes were adjudicated in a multidisciplinary tumor board with recommendations returned to the referring physician.

^68^Ga-DOTATOC PET-CT is shown in this prospective trial to be a test with a high safety profile that is convenient for patients and generates a major change in management in nearly half of the patients who receive it.
